# Two New Eudesmane Sesquiterpenoids from the Flowers of *Chrysanthemum indicum*

**DOI:** 10.1007/s13659-019-0199-9

**Published:** 2019-02-28

**Authors:** Jun-Li Yang, Lei-Lei Liu, Yan-Ping Shi

**Affiliations:** CAS Key Laboratory of Chemistry of Northwestern Plant Resources and Key Laboratory for Natural Medicine of Gansu Province, Lanzhou Institute of Chemical Physics, Chinese Academy of Sciences, Lanzhou, 730000 People’s Republic of China

**Keywords:** *Chrysanthemum indicum*, Sesquiterpenoids, Eudesmane

## Abstract

**Abstract:**

The flowers of *Chrysanthemum indicum*, i.e. Ye-ju-hua recorded in the Chinese Pharmacopoeia, has been widely used in China as an important heat-clearing and detoxifying herb for the treatment of inflammation, headache, and vertigo. A phytochemical investigation of this herb has led to the isolation of two new eudesmane sesquiterpenoids, 7-*epi*-eudesm-4(15),11(13)-diene-1*β*,3*β*-diol (**1**) and 7-*epi*-1*β*-hydroxy-*β*-eudesmol (**2**). The molecular structures of these new sesquiterpenoids were established based on the comprehensive spectroscopic analyses, including NMR, MS, and IR, and comparing with the literatures.

**Graphical Abstract:**

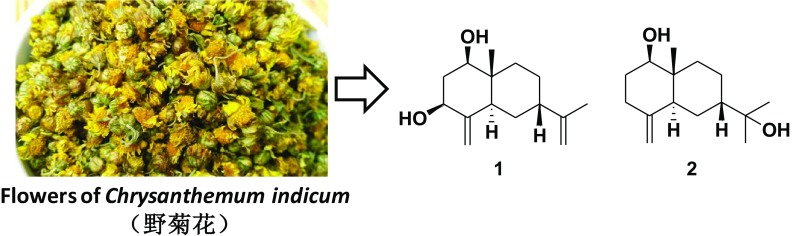

**Electronic supplementary material:**

The online version of this article (10.1007/s13659-019-0199-9) contains supplementary material, which is available to authorized users.

## Introduction

The plants of Asteraceae family, which contains 25 000–30 000 species belonging to over 1000 genera, have been considered as rich sources of eudesmane sesquiterpenoids [1]. Till now there have been over 1000 naturally occurring eudesmane sesquiterpenoids identified from the Asteraceae family, and these plant metabolites possessed diverse oxygenation and cleavage patterns [2]. Eudesmane-type sesquiterpenoids exhibit a wide range of biological activities, such as plant growth regulating, insect antifeedant, antifungal, anti-tumour, antibacterial, and antiviral activities [3–11]. Due to these, eudesmane-type sesquiterpenoids have long been the research focus of the phytochemists and pharmacologists.

**Fig. 1:**
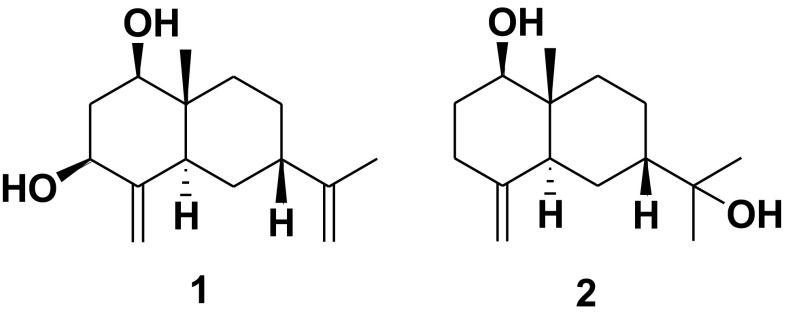
Molecular structures of sesquiterpenoids **1** and **2**

The flowers of *Chrysanthemum indicum* has long been used a traditional heat-clearing and detoxifying herb in China. It is called Ye-ju-hua in the Chinese Pharmacopoeia [12]. The main usage of this traditional Chinese medicine was to treat inflammation, headache, and vertigo and for the preparation of a bitter tea used for antibacterial, antioxidant, and anti-inflammatory purposes [13]. It has long been our goal to find more novel sesquiterpenoids from traditional Chinese herbs [14–18]. As part of this program, an investigation of an aqueous EtOH extract of the flowers of *C. indicum* afforded 2 further new sesquiterpenoids (Fig. 1), 7-*epi*-eudesm-4(15),11(13)-diene-1*β*,3*β*-diol (**1**) and 7-*epi*-1*β*-hydroxy-*β*-eudesmol (**2**). In this paper we will report the procedures for the isolation and structure determination of the two new isolates.

## Results and Discussion

**Fig. 2:**
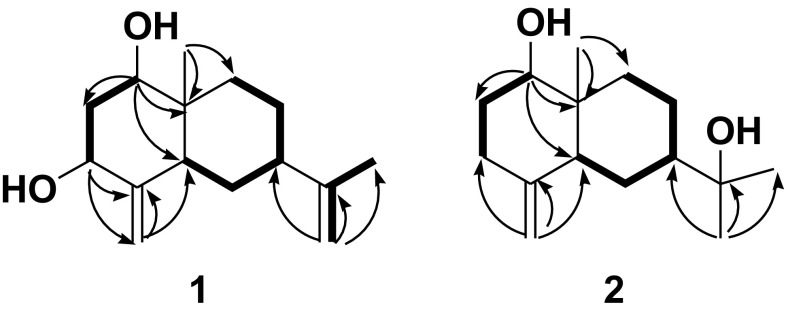
Key COSY (H → H), HMBC (H → C) correlations of **1** and **2**

Sesquiterpenoid **1** was isolated as a white solid with a optical rotation value of $[\alpha ]_{\text{D}}^{20}$ + 5.0 (*c* 0.2, acetone), which suggested this compound could be a racemate. It was assigned a molecular formula of C_15_H_24_O_2_ as determined based on an ion peak at *m/z* 219.1750 [M – H_2_O + H]^+^ (calcd for C_15_H_23_O, 219.1743) in its positive HRESIMS, which suggested four degrees of unsaturation. The IR absorptions at 3384 and 1654 cm^−1^ suggested the presence of hydroxy and olefinic functionalities, respectively. The ^1^H and ^13^C NMR data of **1** revealed (Table 1) an angular methyl (*δ*_H_ 0.74, *δ*_C_ 9.9), a vinyl methyl (*δ*_H_ 1.70, *δ*_C_ 22.2), two oxymethines (*δ*_H_ 3.44, *δ*_C_ 77.1; *δ*_H_ 4.06, *δ*_C_ 70.4), and two terminal double bonds (*δ*_H_ 4.73, 5.11, *δ*_C_ 103.4, 151.6; *δ*_H_ 4.79, 4.91, *δ*_C_ 110.9, 146.4). These observations suggested that **1** possessed a eudesmane framework with two hydroxy groups and two terminal double bonds [14–18]. From the HMBC experiment (Fig. 2), the angular methyl (H_3_-14) correlated with C-1, C-5, C-9 and C-10 (*δ*_C_ 77.1, 38.9, 32.3 and 40.5 respectively), indicating that one hydroxyl group was linked at C-1. Similarly, the exomethylene protons (H_2_-15) correlated with C-3 and C-5 (*δ*_C_ 70.4 and 38.9), which clearly indicated that another hydroxyl group was located at C-3. In the NOE difference experiment, irradiation of H-1 (*δ*_H_ 3.44) enhanced the resonances of H-3 (0.81%), H-2*α* (1.47%), and H-5 (1.15%), but not H_3_-14, indicating that H-1, H-3 and H-5 were all *α*-oriented (assuming that H_3_-14 was *β*-oriented). Comparison of the carbon signal at C-7 (*δ*_C_ 38.4) with that of ligucyperonol (*δ*_C_ 45.1) [19] and *β*-dictyopterol (*δ*_C_ 45.3) [20], suggested a *β*-orientation of H-7. Finally sesquiterpenoid **1** was elucidated as 7-*epi*-eudesm-4(15),11(13)-diene-1*β*,3*β*-diol.

**Table 1:** NMR Spectroscopic Data (in CDCl_3_, 400 MHz) of Sesquiterpenoids **1** and **2**

No.	**1**		**2**	
	^1^H (*δ*)	^13^C (*δ*)	^1^H (*δ*)	^13^C (*δ*)
1	3.44 dd (12.0, 4.4)	77.1 (d)	3.45 dd (11.6, 4.4)	79.3 (d)
2	2.23 m, 1.51 m	40.8 (t)	1.82 m, 1.51 m	31.4 (t)
3	4.06 dd (12.0, 5.2)	70.4 (d)	2.32 m, 2.17 m	34.3 (t)
4		151.6 (s)		149.3 (s)
5	1.73 m	38.9 (d)	2.17 m	41.8 (d)
6	1.85 m, 1.67 m	25.1 (t)	1.73 m, 1.63 m	23.5 (t)
7	2.45 brs	38.4 (d)	1.68 m	41.6 (d)
8	1.88 m, 1.67 m	22.9 (t)	1.78 m, 1.63 m	20.8 (t)
9	1.63 m, 1.37 m	32.3 (t)	1.62 m	34.0 (t)
10		40.5 (s)		39.6 (s)
11		146.4 (s)		74.4 (s)
12	1.70 s	22.2 (q)	1.27 s	29.6 (q)
13	4.91 d (1.6) 4.79 s	110.9 (t)	1.26 s	29.2 (q)
14	0.74 s	9.9 (q)	0.73 s	11.1 (q)
15	5.11 s, 4.73 d (1.6)	103.4 (t)	4.78 d (1.2), 4.54 s	106.8 (t)

Sesquiterpenoid **2** was obtained as a white solid with $[\alpha ]_{\text{D}}^{20}$ + 3.0 (*c* 0.35, acetone), which indicated it maybe exist as a racemate. Its molecular formula was assigned as C_15_H_26_O_2_ on the basis of an ion at m/z 261.1815 [M + Na]^+^ (calcd for C_15_H_26_O_2_Na, 261.1825) in the positive HRESIMS. Its IR absorptions at 3323 and 1645 cm^−1^ indicated the occurrence of hydroxy and olefinic functionalities, respectively. The ^1^H and ^13^C NMR data of **2** demonstrated three methyls (*δ*_H_ 1.11, 1.22, 1.32; *δ*_C_ 11.1, 29.2, 29.6), one oxymethine (*δ*_H_ 3.45; *δ*_C_ 79.3), one oxygenated tertiary carbon (*δ*_C_ 74.4), and one terminal double bond (*δ*_H_ 4.54, 4.78; *δ*_C_ 106.8, 149.3). These observations indicated **2** as an eudesmane sesquiterpenoid possessing two hydroxy groups and one terminal double bond [14–18]. Furthermore, its planer structure was constructed via HMBC experiment as shown in Fig. 2. From the NOE difference experiment, irradiation of H-1 (*δ*_H_ 3.45) enhanced the signals of H-3*α* (1.47%) and H-5 (1.11%) indicating that H-1 and H-5 was *α*-oriented. The chemical shifts of C-1/2/10 of compound **8** (*δ*_C_ 79.3/31.4/39.6 in CDCl_3_), which were similar to those of *β*-dictyopterol (*δ*_C_ 79.3/31.5/40.2 in CDCl_3_) [20], were used to determine the *β*-oriented Me-14. The *β*-orientation of H-7 was deduced from dramatic differences in chemical shifts of C-5/7/9 in compound **8** (*δ*_C_ 41.8/41.6/34.0 in CDCl_3_) and 1*β*-hydroxy-*β*-eudesmol (*δ*_C_ 47.5/48.9/36.9 in CDCl_3_) [21]. Thus, sesquiterpenoid **2** was established as 7-*epi*-1*β*-hydroxy-*β*-eudesmol.

## Experimental Section

### General

Optical rotations were measured on a Perkin-Elmer model 341 polarimeter with a 1 dm cell. IR spectra were obtained on a Nicolet NEXUS 670 FT-IR spectrometer. NMR spectra were recorded on a Bruker AVANCE Ш-400 spectrometer. Chemical shifts are given as *δ* (ppm) using TMS as internal standard. HRESIMS were carried out on a Bruker APEX II mass spectrometer. Sephadex LH-20 (GE Healthcare Bio-science AB, Sweden), C_18_ reversed-phase silica gel (YMC, ODS-A, AA120S50), Silica gel (200**–**300 mesh, Qingdao Marine Chemical factory, Qingdao, China.) were used for column chromatography (CC). TLC using silica GF_254_ (10**–**40 *μ*m) was detected at 254 nm and spots were visualized by spraying with 5% H_2_SO_4_ in EtOH (v/v) followed by heating.

### Plant Material

The flowers of *C. indicum* was purchased from Huanghe Medicinal Material Market in Gansu in 2009 and identified by Associate Prof. H.Y. Qi of CAS Key Laboratory of Chemistry of Northwestern Plant Resources in LICP, where a voucher specimen (No. ZY2009C001) was deposited.

### Extraction and Isolation

The air-dried flowers of *C. indicum* (2.0 kg) were pulverized and extracted with 95% EtOH (3 × 5 L) at 50 °C for 3 h each time. The combined extracts were evaporated to dryness under reduced pressure. The resulting residue was mixed with H_2_O (1.5 L) to form a suspension and partitioned successively with petroleum ether (PE), EtOAc, and *n*-BuOH. The PE-soluble part (60 g) was subjected to silica gel CC with a gradient system of PE–EtOAc (60:1, 30:1, 15:1, 10:1, 7:1, 5:1, 3:1 and 1:1) to afford eight fractions (A–H) based on TLC analysis. Fractions F–G was purified over a column of Sephadex LH-20 using CHCl_3_–MeOH (2:1) to give tho sub-fractions (F1 and G1) without pigments. Sub-fraction F1 was separated by silica gel CC eluted repeatedly with CHCl_3_–EtOAc (25:1, 15:1 and 10:1), PE–EtOAc (10:1), CHCl_3_–Me_2_CO (15:1), and a column of C_18_ reversed-phase silica gel (MeOH–H_2_O, 70:30, 60:40), to give **2** (12.3 mg). Sub-fraction G1A was repeated purified by silica gel eluted with CHCl_3_–Me_2_CO (10:1) and PE–Me_2_CO (8:1.5), and then over a C_18_ reversed-phase silica gel eluted by (MeOH–H_2_O, 60:40, 50:50) to yield **1** (16.1 mg).

### Characteristic Data of Compounds

7-*epi*-eudesm-4(15),11(13)-diene-1*β*,3*β*-diol (**1**): White solid; $[\alpha ]_{\text{D}}^{20}$ + 5.0 (*c* 0.2, acetone); IR (KBr) *ν*_max_: 3384, 2924, 1654, 1458, 1064, 803 cm^−1^; ^1^H and ^13^C NMR data, Table 1; HRESIMS: *m/z* 219.1750 [M – H_2_O + H]^+^ (calcd for C_15_H_23_O, 219.1743).

7-*epi*-1*β*-hydroxy-*β*-eudesmol (**2**): White solid; $[\alpha ]_{\text{D}}^{20}$ + 3.0 (*c* 0.35, acetone); IR (KBr) *ν*_max_: 3323, 2970, 1645, 1455, 1034, 884 cm^−1^; ^1^H and ^13^C NMR data, Table 1; HRESIMS: *m/z* 261.1815 [M + Na]^+^ (calcd for C_15_H_26_O_2_Na, 261.1825).

## Electronic supplementary material

Below is the link to the electronic supplementary material.

Supplementary material 1 (DOC 5619 kb)
