# Supplementary material for: Two New Eudesmane Sesquiterpenoids from the Flowers of Chrysanthemum indicum
Source: Nat Prod Bioprospect. 2019 Feb 28;9(2):145–8. doi: 10.1007/s13659-019-0199-9 (PMC6426898; doi:10.1007/s13659-019-0199-9)
Supplement: Supplementary file 1 — Supplementary material 1 (DOC 5619 kb) [file 13659_2019_199_MOESM1_ESM.doc]

**Two New Eudesmane Sesquiterpenoids from the Flowers of *Chrysanthemum indicum***

Jun-Li Yang, Lei-Lei Liu, and Yan-Ping Shi [[1]](#footnote-2)*

CAS Key Laboratory of Chemistry of Northwestern Plant Resources and Key Laboratory for Natural Medicine of Gansu Province, Lanzhou Institute of Chemical Physics, Chinese Academy of Sciences, Lanzhou 730000, P. R. China

**For Sesquiterpenoid 1**

^1^H NMR spectrum of Sesquiterpenoid **1** in CDCl_3_.


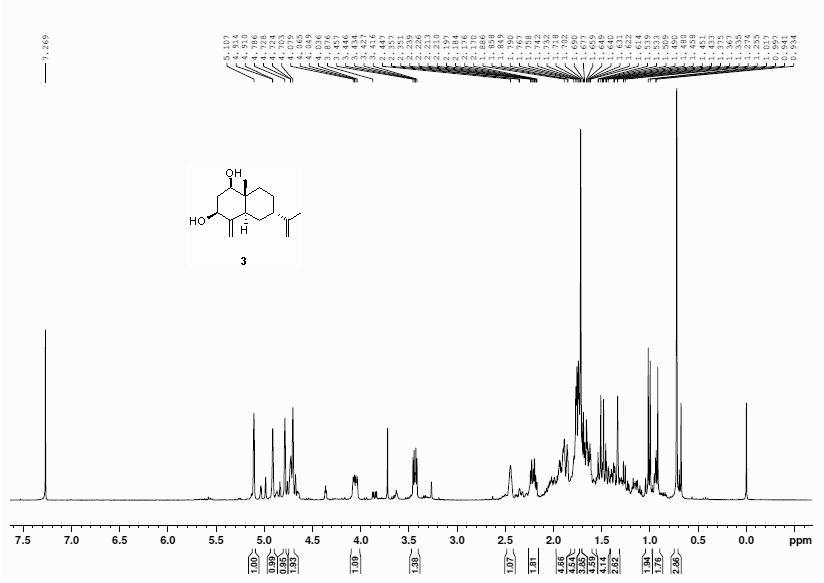


^13^C NMR and DEPT spectra of Sesquiterpenoid **1** in CDCl_3_.


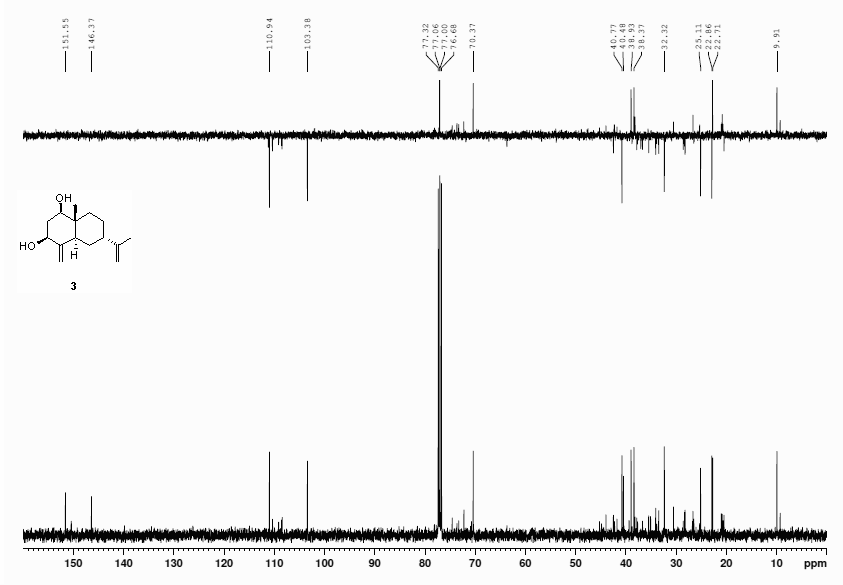


^1^H-^1^H COSY spectrum of Sesquiterpenoid **1** in CDCl_3_.


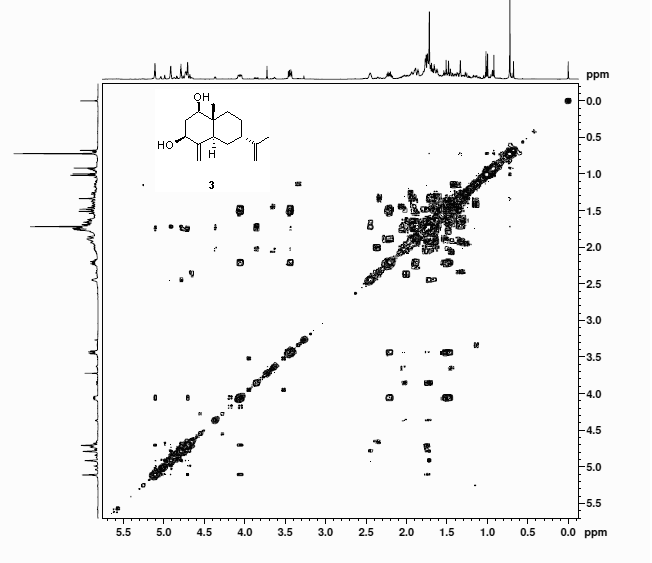


HSQC spectrum of Sesquiterpenoid **1** in CDCl_3_.


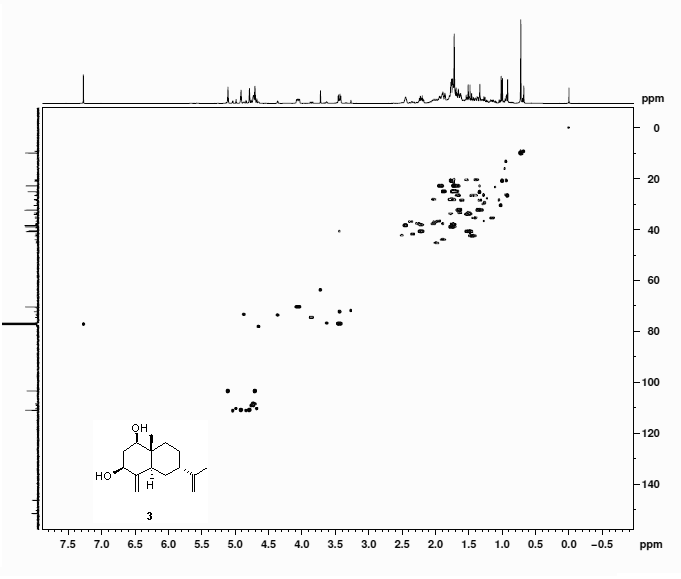


HMBC spectrum of Sesquiterpenoid **1** in CDCl_3_.


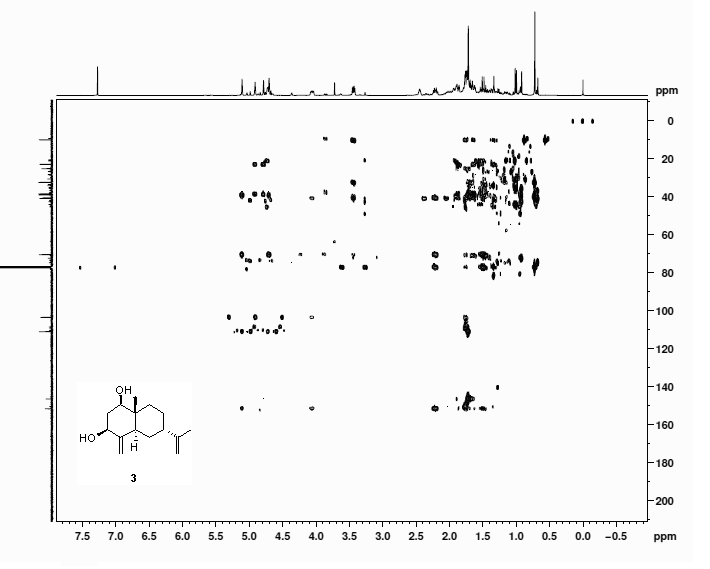


NOEs difference spectra of Sesquiterpenoid **1** in CDCl_3_.


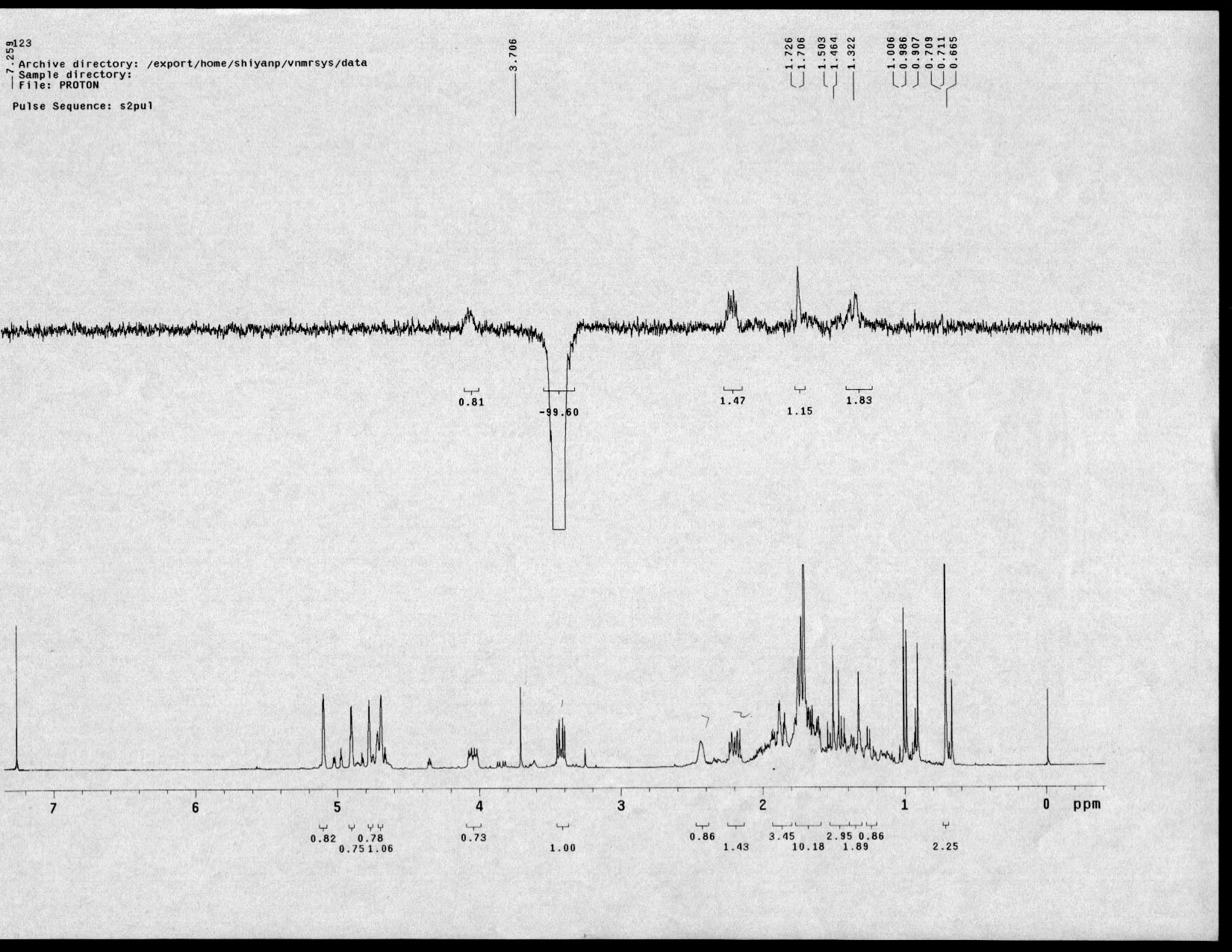


NOEs difference spectra of Sesquiterpenoid **1** in CDCl_3_.


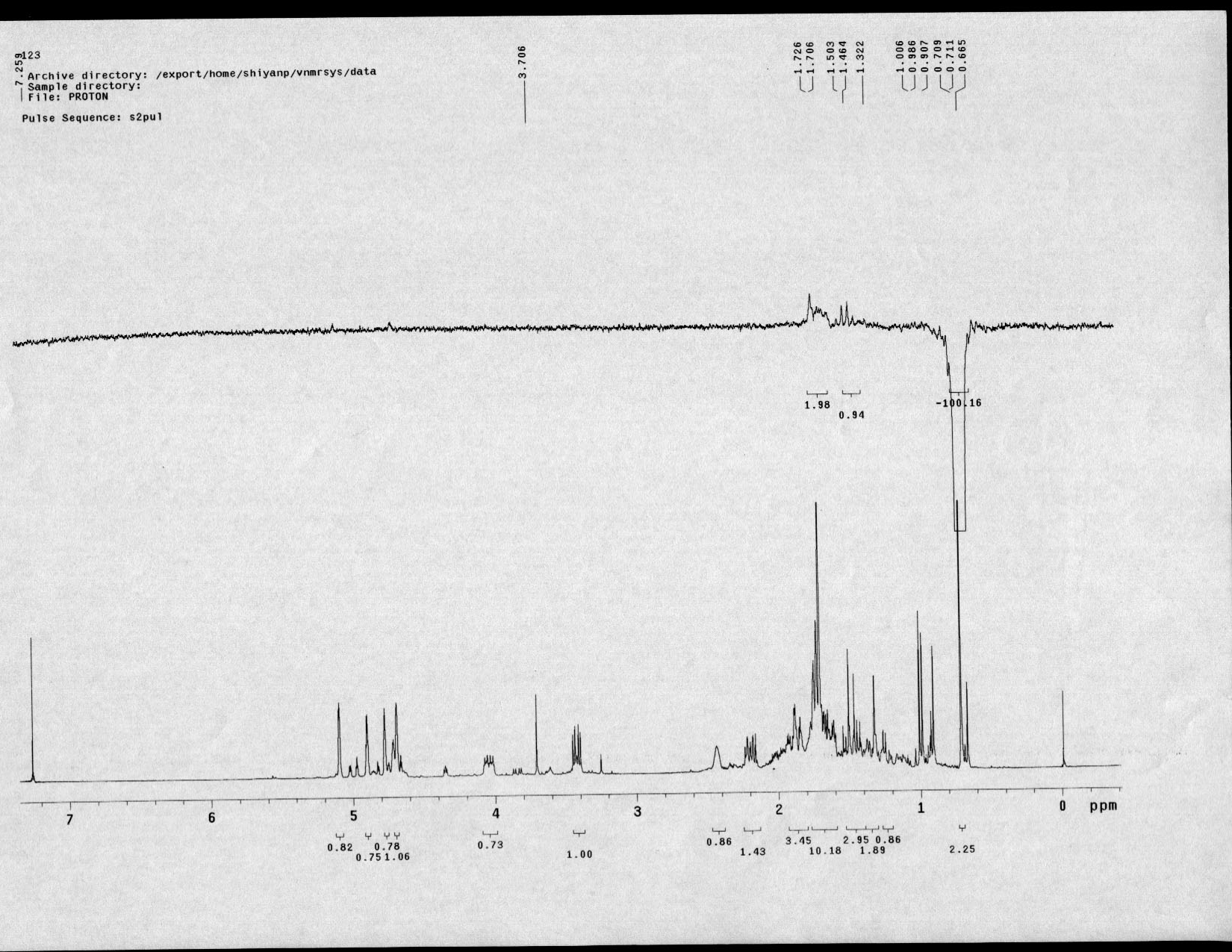


**For Sesquiterpenoid 2**

^1^H NMR spectrum of Sesquiterpenoid **2** in CDCl_3_.


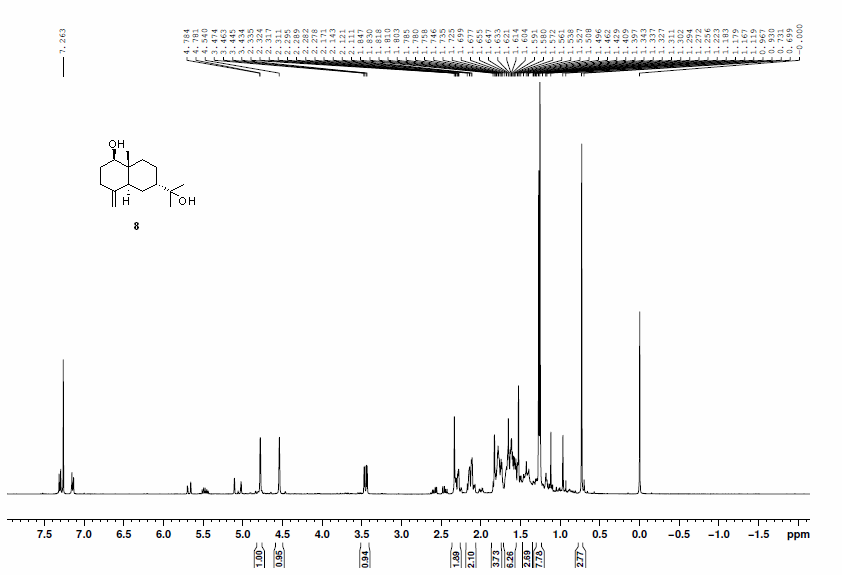


^13^C NMR and DEPT spectra of Sesquiterpenoid **2** in CDCl_3_.


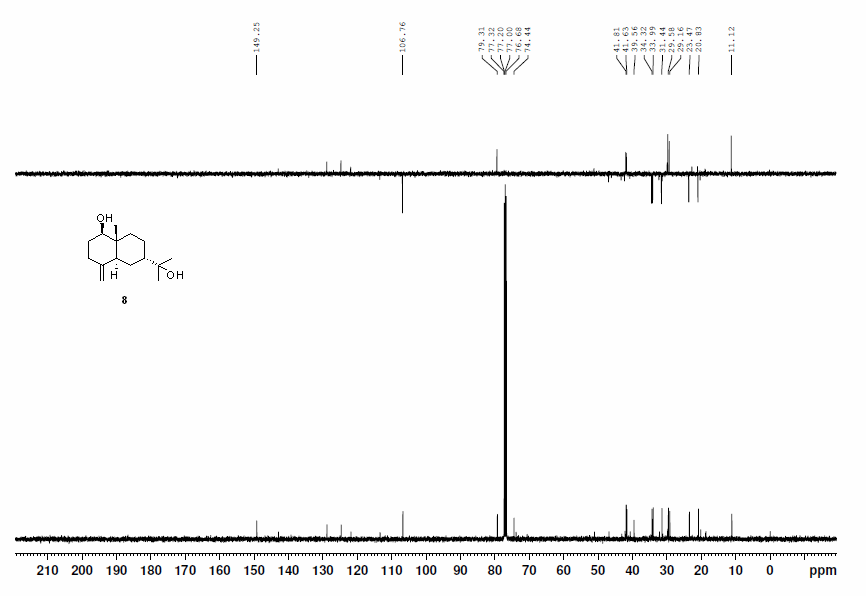


^1^H-^1^H COSY spectrum of Sesquiterpenoid **2** in CDCl_3_.


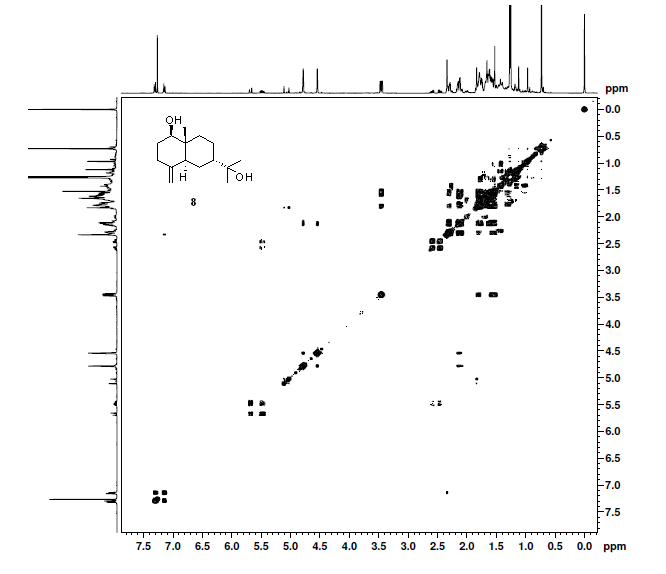


HSQC spectrum of Sesquiterpenoid **2** in CDCl_3_.


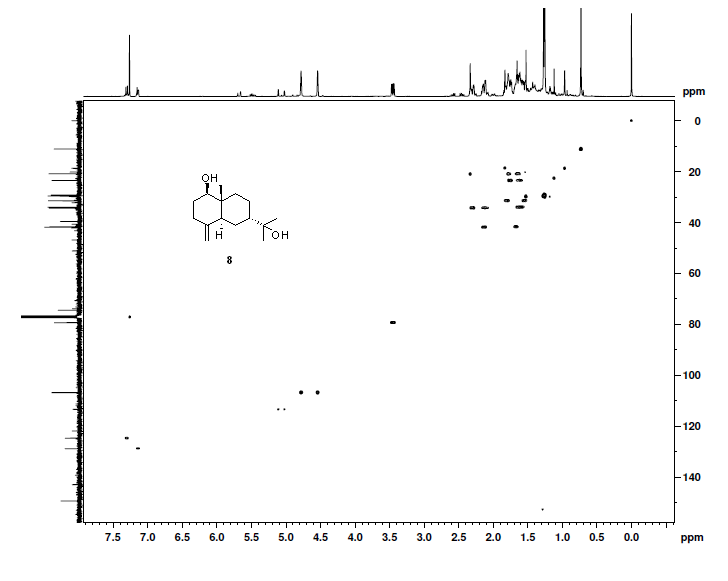


HMBC spectrum of Sesquiterpenoid **2** in CDCl_3_.


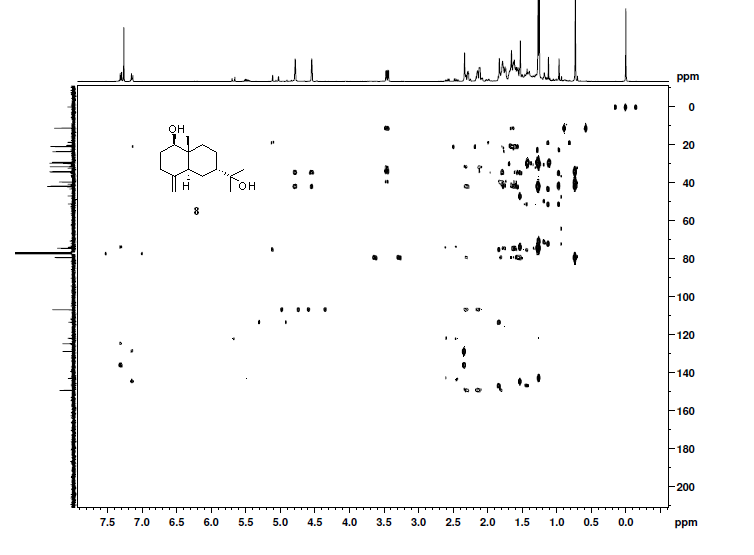


NOEs difference spectrum of Sesquiterpenoid **2** in CDCl_3_.


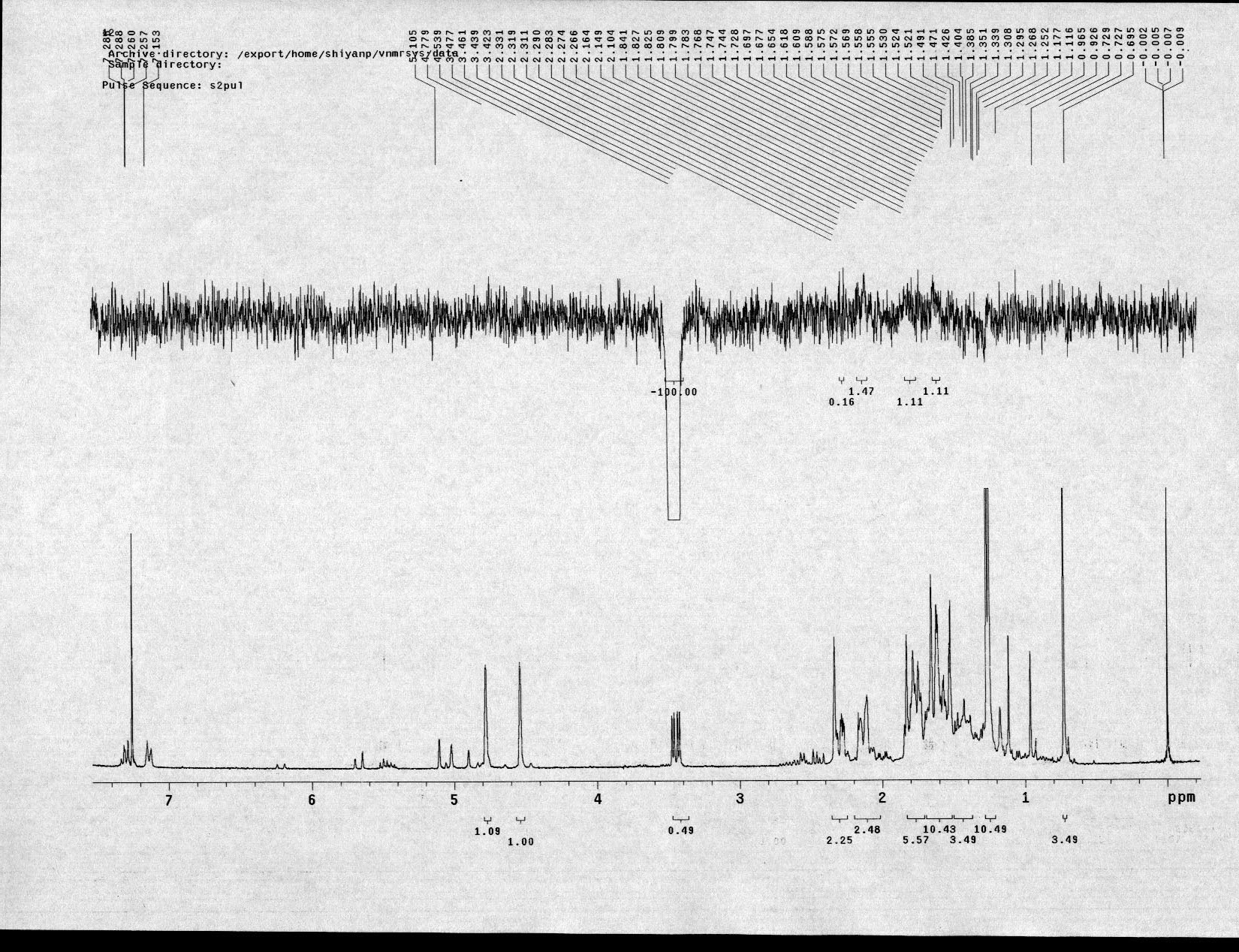


NOEs difference spectrum of Sesquiterpenoid **2** in CDCl_3_.


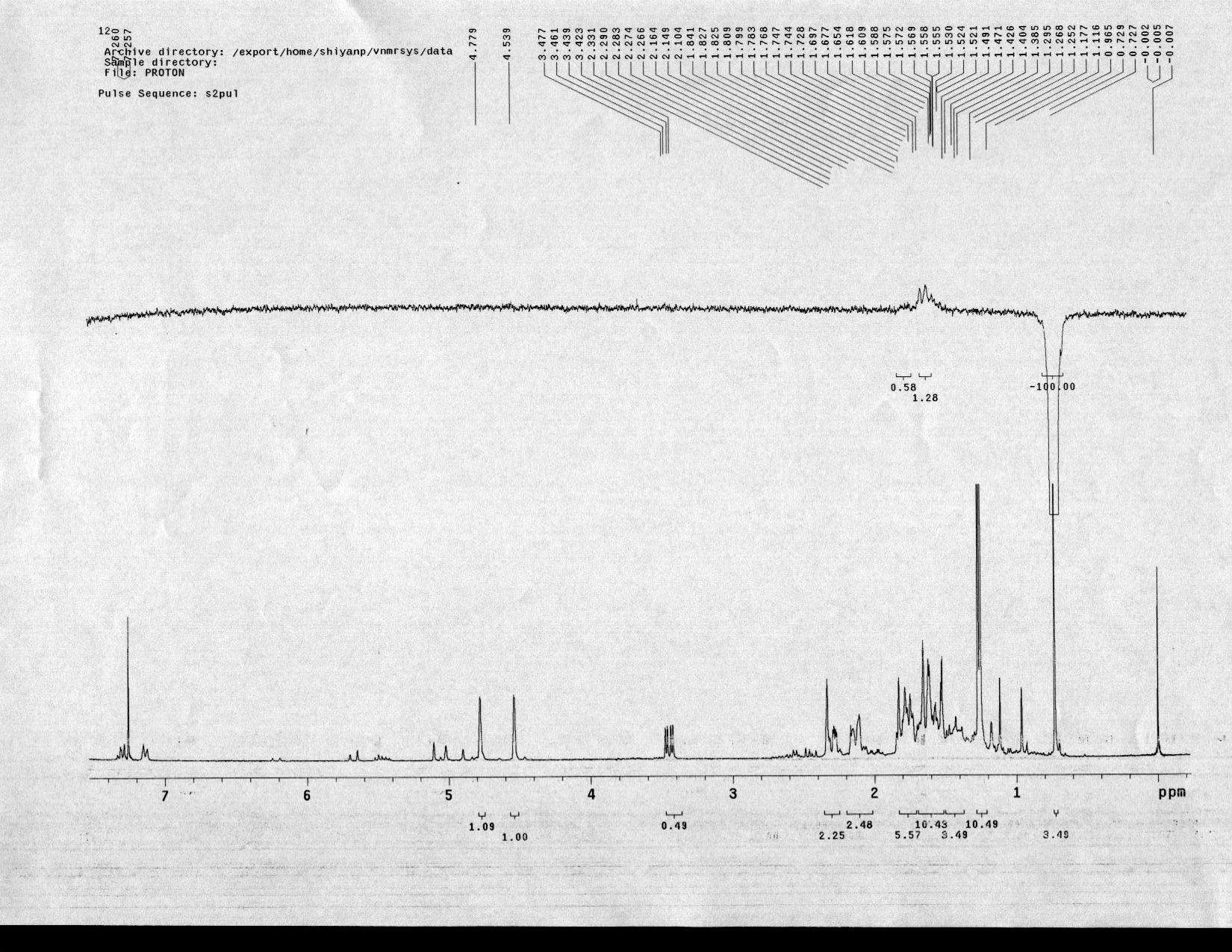


1. ^^ To whom correspondence should be addressed. Tel: +86-931-4968208.

   Fax: +86-931-4968094. E-mail: [shiyp@licp.cas.cn](mailto:shiyp@licp.cas.cn) (Y.-P. Shi) [↑](#footnote-ref-2)
